# Complete Genome Sequence of Proteus mirabilis Siphophage Stubb

**DOI:** 10.1128/MRA.01185-19

**Published:** 2019-10-24

**Authors:** Taylor A. Bourgeois, Lauren Lessor, Chandler O’Leary, Rohit Kongari, Mei Liu

**Affiliations:** a Center for Phage Technology, Texas A&M University, College Station, Texas, USA; DOE Joint Genome Institute

## Abstract

Proteus mirabilis, a Gram-negative bacterium belonging to the family *Enterobacteriaceae*, is a common cause of urinary tract infections. Phages infecting Proteus mirabilis could be used as therapeutics to treat infections caused by this bacterium. This announcement describes the complete genome sequence of the T5-like P. mirabilis phage Stubb.

## ANNOUNCEMENT

Proteus mirabilis is a Gram-negative pathogen that often causes symptomatic infections, such as cystitis and pyelonephritis, and asymptomatic bacteriuria (1, 2). With the reportedly increased rate of antibiotic resistance among P. mirabilis strains (3) and the promising *in vitro* treatment results of catheters with phage cocktails (4), phage therapy against P. mirabilis may prove useful in treating infections caused by this bacterium.

Phage Stubb was isolated using a P. mirabilis strain from the municipal wastewater treatment plant in Brazos County, TX, in 2013. Host bacteria were cultured on nutrient broth or agar (Difco) at 37°C with aeration. Phages were isolated and propagated by the soft-agar overlay method (5). It was identified as a siphophage using negative-stain transmission electron microscopy performed at the Texas A&M University Microscopy and Imaging Center, as described previously (6). Phage genomic DNA was prepared using a modified Promega Wizard DNA cleanup kit protocol (6). Pooled indexed DNA libraries were prepared using the Illumina TruSeq Nano low-throughput (LT) kit, and the sequence was obtained from the Illumina MiSeq platform using the MiSeq V2 500-cycle reagent kit following the manufacturer’s instructions, producing 1,069,908 paired-end reads for the index containing the phage genome. The quality of the reads was checked in FastQC 0.11.5 (https://www.bioinformatics.babraham.ac.uk/projects/fastqc/), and the reads were trimmed with FastX toolkit 0.0.14 (http://hannonlab.cshl.edu/fastx_toolkit/download.html) and assembled in SPAdes 3.5.0 (7). The assembled genome was closed by PCR using primers (5′-TAGTAGTGGGTATGTTTGTTGG-3′ and 5′-ACTAAATGCCCACATCAGTAG-3′) facing off the ends of the assembled contig, and Sanger sequencing of the resulting product was conducted with the contig sequence manually corrected to match the Sanger sequencing read. Protein-coding genes were predicted using GLIMMER 3.0 (8) and MetaGeneAnnotator 1.0 (9) and corrected manually if needed. The tRNA genes were predicted using ARAGORN 2.36 (10). Rho-independent termination sites were identified via TransTerm (http://transterm.cbcb.umd.edu/). Sequence similarity searches were done by BLASTp 2.2.28 (11), with a maximum expectation cutoff of 0.001 against the NCBI nonredundant (nr), UniProt Swiss-Prot (12), and TrEMBL databases. InterProScan 5.15-54.0 (13), LipoP (14), and TMHMM v2.0 (15) were used to predict protein function. HHpred with ummiclust30_2018_08 for multiple sequence alignment (MSA) generation and PDB_mmCIF70 for modeling in the HHsuite v3.0 release were also used for functional prediction (16). All analyses were conducted at default settings via the Center for Phage Technology (CPT) Galaxy (17) and WebApollo (18) interfaces (https://cpt.tamu.edu/galaxy-pub).

*Proteus* phage Stubb was assembled at 27.8-fold coverage to a single contig of 104,410 bp, with only one copy of a 11,415-bp direct terminal repeat determined by PhageTerm (19). It has a G+C content of 38.5%. The progressiveMauve program (20) was used to compare Stubb nucleotide similarity against the NCBI nr/nucleotide database, and the most similar organism at 83.9% identity was *Proteus* phage PM135 (GenBank accession no. NC_042090). Despite having a nucleotide similarity of only 19.8% with T5 (GenBank accession no. AY543070), the Stubb genome appears to be syntenic with the T5 genome (21). Most of the genes with a predicted function were involved in phage morphogenesis and in DNA replication. Lysis genes encoding a class III holin and endolysin with a hydrolase domain and an overlaping spanin pair were identified.

### Data availability.

The genome sequence of phage Stubb was deposited under GenBank accession no. MH830339. The associated BioProject, SRA, and BioSample accession numbers are PRJNA222858, SRR8771457, and SAMN11234224, respectively.
